# Supplementary material for: Complexome profiling of the Chlamydomonas psb28 mutant reveals TEF5 as an early PSII assembly factor
Source: Plant Cell. 2025 Mar 17;37(6):koaf055. doi: 10.1093/plcell/koaf055 (PMC12164590; doi:10.1093/plcell/koaf055)
Supplement: koaf055_Supplementary_Data [file koaf055_supplementary_data.zip › psb28_interactive.zip!/plots/intensity/qualityPlots/peptides/PKS1_Cre10.g449750.t1.1.html]

### Cre10.g449750.t1.1

Trivial name: PKS1  
SeqCoverage [median]: 0.10  
Mass: 2336.70 kDa  
  
 

| Sequence | MissedCleavage | Mass [Da] | wtIntensity | muIntensity | Score |
| --- | --- | --- | --- | --- | --- |
| LAPAAHAAPTVAPLPSRR | 1 | 1795.02 | 0.00 | 794.10 | 15.39 |
| VAALSPDGRCK | 1 | 1115.58 | 0.00 | 274.11 | 49.30 |
|  |  |  | 0.0 | 1068.2 |  |
